# Over-Expression of Endogenous SUGARWIN Genes Exalted Tolerance against *Colletotrichum* Infection in Sugarcane

**DOI:** 10.3390/plants10050869

**Published:** 2021-04-26

**Authors:** Aqsa Parvaiz, Ghulam Mustafa, Muhammad Sarwar Khan, Muhammad Amjad Ali

**Affiliations:** 1 Center of Agricultural Biochemistry and Biotechnology (CABB), University of Agriculture, University Road, Faisalabad 38040, PB, Pakistan; aqsapervaiz333@gmail.com (A.P.); sarwarkhan_40@hotmail.com (M.S.K.); 2 Department of Plant Pathology, University of Agriculture, University Road, Faisalabad 38040, PB, Pakistan; amjad.ali@uaf.edu.pk

**Keywords:** nuclear transformation, sugarcane, young leaf whorls, SUGARWIN, selection marker, phosphinothricin, maize ubiquitin promoter, *Colletotrichum falcatum*

## Abstract

Sugarcane being the major contributor of sugar and potential source of biofuel around the globe, occupies significant commercial importance. Red rot is the most devastating disease of sugarcane, severely affecting its quality as well as yield. Here we report the overexpression of *SUGARWIN1* and *SUGARWIN2* genes in any field crop for the first time. For this purpose, *SUGAWIN1* and *SUGARWIN2* were cloned downstream of maize ubiquitin (*Ubi-*1) promoter to construct two independent expression cassettes. The *bar* gene conferring resistance against phosphinothricin was used as selectable marker. Embryogenic calli of sugarcane were bombarded with both expression cassettes and selected on regeneration medium supplemented with phosphinothricin. The phosphinothricin-resistant shoots were rooted and then, analyzed using molecular tools at the genomic as well as transcriptomic levels. The transcriptomic analysis, using real time qPCR, showed that expression of *SUGARWIN1* (SWO) and *SUGARWIN2* (SWT) was higher in transgenic plants as compared to untransformed plants. Our results further demonstrated that over expression of these genes under maize ubiquitin (*Ubi*-1) promoter causes significant restriction in proliferation of red rot causal agent, *Colletotrichum falcatum* in sugarcane transgenic plants, under in vitro conditions. This report may open up exciting possibilities to extend this technology to other monocots for the development of crops with better ability to withstand fungal pathogens.

## 1. Introduction

Fungal diseases bring about substantial yield losses in sugarcane crop. More than 160 fungal pathogens are known to harm sugarcane, while seven diseases with new etiology have been renowned and reported [1]. Among these, the most noxious one is red rot, also famous as “cancer” of sugarcane [2], which is triggered by *Colletotrichum falcatum* Went. Quality and quantity of the cane is severely affected by this disastrous red rot; it reduces cane weight up to 29% and sugar recovery up to 30% [3]. Red rot is one of the ancient diseases of sugarcane in several countries, including the United States, Thailand, Taiwan, Bangladesh, and Pakistan [4,5]. Cane stalk may be infected by red rot at both initial as well as mature stages of growth. Some common symptoms include discoloration, in addition to pathogen produced invertases, which hydrolyze the sucrose into fructose and glucose and cause dryness of the cane stalk. Hence, vegetative growth of the plants is halted [6]. Red rot disease can be eradicated by adopting various methods of disease management, including tissue culture, breeding, biological, and chemical control. All above-mentioned methods have several boundaries and restrictions, i.e., biological methods (growth-promoting bacteria) [7] show capricious results under field condition. Similarly, chemical methods are expensive and pollute the environment. While somaclonal variations are linked with the selection of fungus-resistant cells [8,9], disease resistant varieties can be developed by breeding [10], but breeding is arduous and tedious, especially in case of sugarcane. All these restrictions and limitations may be conquered by genetic modification of sugarcane plants. Particular genes can be expressed into the sugarcane genome to confer resistance against *Colletotrichum falcatum* [11]. Hence, it can be said that transgenic technology is the only high-tech approach that has ability to tackle all of the above-mentioned obstacles through the development of environmentally friendly genotypes, having a wide range of resistance against plant pathogens including fungi [12].

*SUGARWIN1* and *SUGARWIN2* are class II chitinases belonging to PR-4 family and have a higher level of similarity with antifungal BARWIN, a barley wound-inducible protein [13]. Several plant species including *Nicotiana tabacum*, *Hevea brasiliensis*, *Triticum aestivum*, and *Solanum lycopersicum* have proteins with a BARWIN-like domain, either with or without chitin binding domains [14,15]. SUGARWIN2 protein has antifungal activity against *Colletotrichum falcatum* [16] and *Fusarium verticillioides* [13]. Additionally, SUGARWIN2 has been documented to have antifungal activity against the pathogenic fungus, *Ceratocystis paradoxa* but does not have antifungal activity against nonpathogenic fungi, i.e., *Aspergillus nidulans* and *Saccharomyces cerevisiae* [16]. SUGARWIN2 has also been reported to affect the viability and morphogenesis of fungi by PCD (programmed cell death) followed by overflow of intracellular material thus increasing the point of fractures and vacuolization [13,16].

Considering the importance of *SUGARWIN* genes against fungal infection, these genes were overexpressed in sugarcane under constitutive promotors (maize ubiquitin) to enhance the resistance against red rot causal agent, *Colletotrichum falcatum*.

## 2. Results

### 2.1. Callus Induction and Regeneration

**Figure 1:**
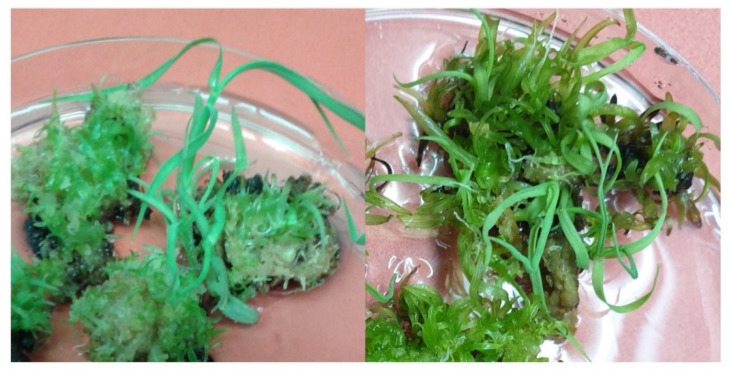
Depiction of massive shoot production from the embryogenic calli proliferated from the whorls of young undifferentiated leaves of genotype SPF-234. Maximum shoot induction was attained on MS medium supplemented with 0.5 mg/L 6-Benzylaminopurine and cultures incubated in 16 hrs light + 8 hrs dark regime at 25 ± 1 °C.

Young, unfurled leaves of sugarcane genotype SPF-234 were sliced into 1.5–2.0 mm thick discs and were placed on callus induction medium, augmented with 2,4-D (3 mg/L). Approximately 21–28 days old calli were shifted on regeneration media supplemented with different concentrations (0 mg/L, 0.5 mg/L, 1.0 mg/L, 1.5 mg/L, and 2.0 mg/L) of 6-Benzylaminopurine (BAP) and the highest count of shoots were found on 0.5 mg/L BAP (Figure 1).

### 2.2. Development of Plant Expression Vectors

**Figure 2:**
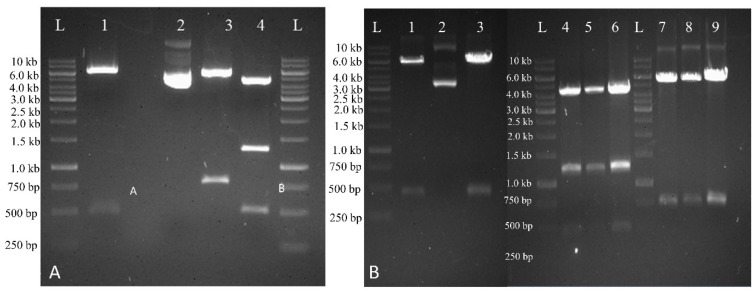
Confirmation of final transformation vectors harboring synthetic *SUGARWIN1* and *SUGARWIN2* genes. (**A**) Restriction analysis of *SUGARWIN1* gene construct. Lane 1 represents restriction with *Spe*I and *Bam*HI (fragment of 500 bp), Lane 2 represents undigested plasmid DNA, Lane 3 shows restriction with *Spe*I and *Not*I (fragment of 780 bp)*,* Lane 4 shows restriction with *Spe*I and *Mlu*I (fragments of 470 and 1400 bp whereas L represents 1 kb DNA ladder. **(B)** Restriction analysis of *SUGARWIN2* gene construct. Lanes 1 and 3 represent restriction with *Spe*I and *Bam*HI (fragment of 480 bp), Lane 2 shows undigested plasmid DNA, Lanes 4, 5, 6 represent restriction with *Spe*I and *Mlu*I (fragments of 470 bp and 1400 bp) whereas Lanes 7, 8, 9 show restriction with *Spe*I and *Not*I (fragments of 780 bp).

SUGARWIN1 and SUGARWIN2 proteins are involved in helping out plants to withstand biotic stress conditions. The synthetic genes *SUGARWIN1* and *SUGARWIN2*, with desired restriction sites *Bam*HI at the 3′-end and *Spe*I at the 5′-end were cloned in pUC19 vector at *PstI* and *KpnI* restriction sites. Then synthetic genes were excised from pUC19 using *Bam*HI and *Spe*I restriction enzymes and ligated into pUbiAB vector under maize ubiquitin (*Ubi-1*) promoter and *nos* terminator. The *bar* gene conferring resistance against herbicide glufosinate or phosphinothricin was used as selectable marker gene. It was cloned under *CaMV 35S* promoter and terminator and was physically linked with the aforementioned recombinant genes. Physical linkage between selection cassette and expression cassette is of pivotal importance to effectively transform plant cells. It increases the transformation efficiency by promoting growth of transformed cells in selection media, thus growth of untransformed cells is suppressed. All recombinant genes were cloned separately, and two independent constructs were developed. The final transformation vectors were confirmed by restriction analysis using various combinations of restriction endonucleases (Figure 2).

### 2.3. Plant Nuclear Transformation and Recovery of Transgenes

**Figure 3:**
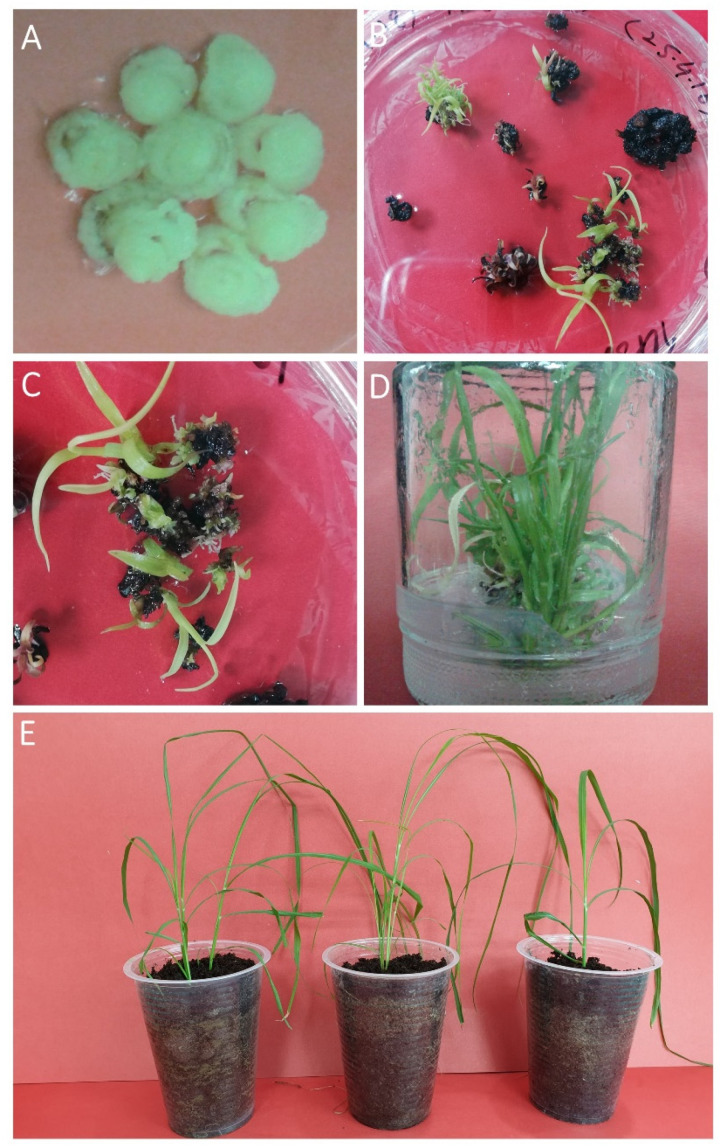
Selection and screening of putative sugarcane transformants. (**A**) Depicts 21–28 days old sugarcane calli proliferated in complete dark conditions. (**B**) Depicts bombarded calli on selection medium. Only transformed cells endured the selection pressure and were able to regenerate, whereas untransformed cells died. (**C**) Close up view of some shoots from figure B exhibiting resistance against phosphinothricin. (**D**) Resistant shoots were shifted to root induction medium. (**E**) Acclimatized transgenic sugarcane plants.

Particle bombardment has been used for gene delivery since 1992 [17,18,19] and is a reliable method for DNA delivery into the cells/tissues. Selection of transformants is based on some selectable marker gene (*bar*), which provides resistance against phosphinothricin. Kill curve was developed in order to find the minimum lethal dose of phosphinothricin for the screening and selection of putative transgenic cells [20,21]. An amount of 3 mg/L phosphinothricin was determined as the minimum lethal dose using kill curve method for optimal screening and selection. Then, 21–28 days-old calli were co-bombarded using the Biolistic^®^ PDS-1000/He particle delivery system (Bio-Rad, Hercules, CA, USA) following the established protocol. Then, the bombarded sugarcane calli were kept in the dark for two days, followed by sub-culture on regeneration medium having 3 mg/L phosphinothricin and were incubated in the light (16 h/8 h photoperiod (3000–4000 lux day intensity)) at 25 ± 1 °C. Only the transformed cells were able to survive, whereas un-transformed cells appeared to be turned brown followed by death. The sugarcane shoots appearing on selection medium (regeneration medium (RM) with 3 mg/L phosphinothricin) were shifted to phosphinothricin having MS0 medium in glass jars for root formation (Figure 3).

### 2.4. Tracking Transgene Integration through Ploymerase Chain Reaction (PCR)

**Figure 4:**
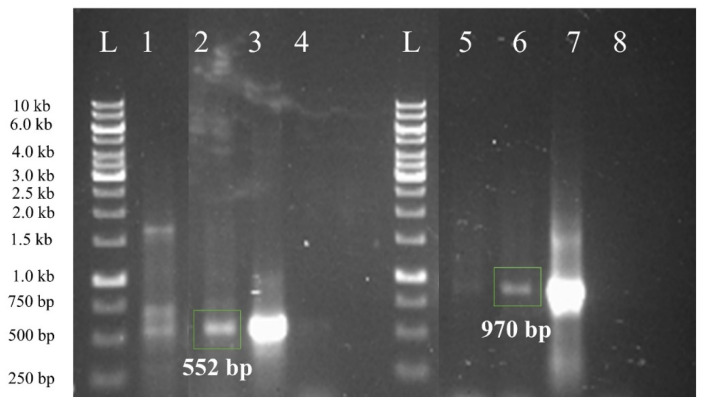
Confirmation of transgene integration with polymerase chain reaction (PCR). L represents 1 kb DNA ladder, lanes 1 and 2 represent amplification of *bar* gene from transgenic sugarcane, while lane 3 represent amplification of *bar* gene from plasmid DNA (positive control). Lane 4 represent amplification from untransformed sugarcane plants (negative control). Similarly, lanes 5 and 6 represent amplification of *SUGARWIN* genes along with its promoter and terminator from putative transformants of sugarcane while lane 7 showed amplification of *SUGARWIN* gene along with its promoter and terminator from plasmid DNA. Lane 8 represented amplification from sugarcane wild type untransformed plant DNA.

Confirmation of transgenic plants using molecular tools is a decisive step of genetic transformation protocols. The resistant shoots were evaluated to confirm transgene integration into the sugarcane genome using two sets of PCR primers. One set of primers flanking *bar* gene while other set of primers with forward primer flanking promoters and terminators of *SUGARWIN* gene constructs, were used. Amplification of a fragment of 552 bp, confirmed integration of the *bar* gene into sugarcane genome, however no amplification was observed in untransformed (control) plants (Figure 4). Similarly, promoter-terminator primer pair resulted in amplification of a fragment of 970 base pairs for both of the constructs (Figure 4). These results confirmed the integration of transgenes into sugarcane genome.

### 2.5. Tracking Transgene Expression by RT-qPCR

**Figure 5:**
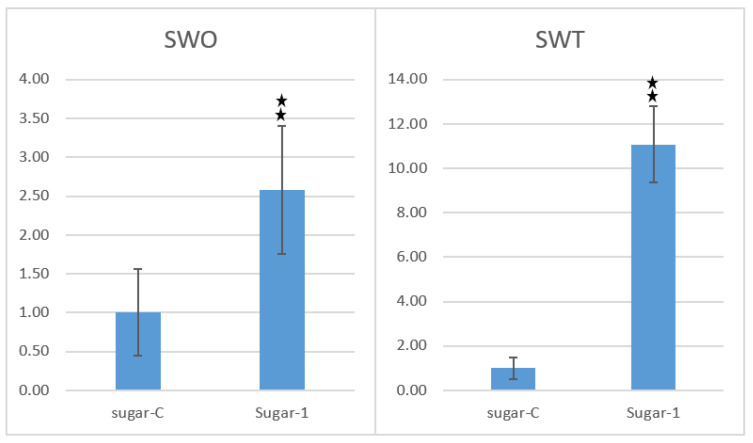
Expression analysis of *SUGARWIN1* and *SUGARWIN2* in putative transgenic plants through qPCR. *SUGARWIN1* (SWO) and *SUGARWIN2* (SWT) expression was evaluated in transgenic (Sugar-1) as well as control (sugar-C) sugarcane plants growing under controlled conditions at 25 ± 1 °C. Data from three independent replicates were analyzed with the *t*-test; ns = not significant; 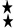 *p* = 0.01.

Real time qPCR is a quick, specific, and highly sensitive tool for the tracking and quantification of transcriptome [22]. Total cellular RNA was isolated by GeneJET^TM^ plant RNA purification mini kit (ThermoFisher Scientifc, Waltham, MA, USA) from untransformed as well as PCR-positive transgenic sugarcane plants growing in the growth room at 25 ± 1 °C. Synthesis of cDNA was carried out using RevertAid First Strand cDNA synthesis kit following manufacturer’s protocol and was used as template in qPCR reaction to evaluate the expression level of *SUGARWIN1* and *SUGARWIN2* genes in transgenic plants in comparison with control plants (non-transgenic plants of same sugarcane genotype). CFX96™ Real-Time PCR Detection System (Bio-Rad, Hercules, CA, USA) was used to perform expression analysis. Reaction conditions were normalized using *GAPDH* and *Actin* as reference genes. Results of real time qPCR showed that expression of *SUGARWIN1* (SWO) and *SUGARWIN2* (SWT) was higher in transgenic plants, as compared to untransformed ones (Figure 5). Hence, recombinant *SUGARWIN1* and *SUGARWIN2* are being over-expressed in the transgenic plants. Data from three independent replicates were analyzed using the *t*-test.

### 2.6. Fungal Bioassay to Assess Anti-Pathogenic Activity of Putative Transgenic Plants

**Figure 6:**
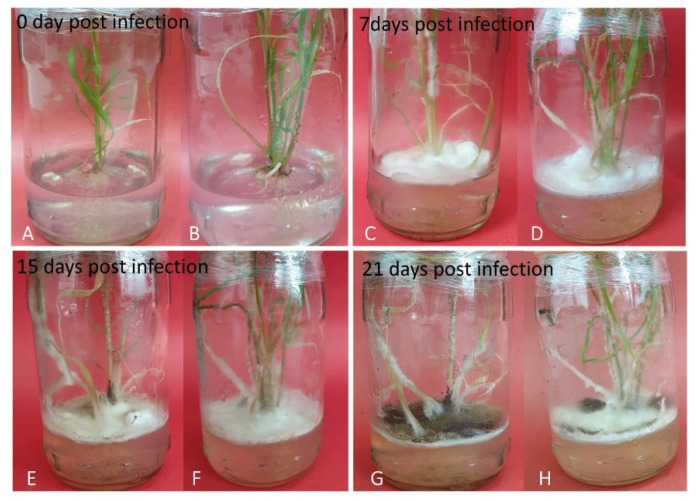
Fungal bioassay of the transgenic sugarcane lines in order to assess their anti-pathogenic activity. Tranformed and untransformed sugarcane plants were infected with *Colletotrichum falcatum* under in vitro conditions. (**A**,**C**,**E**,**G**) Untransformed sugarcane plants at 0, 7, 15, and 21 days after infection with *Colletotrichum falcatum*. (**B**,**D**,**F**,**H**) Transgenic sugarcane plants at 0, 7, 15, and 21 days after infection with *Colletotrichum falcatum*.

Putative transgenic plants were further analyzed to assess the anti-pathogenic activity of synthetic *SUGARWIN1* and *SUGARWIN2* genes. Transgenic and wildtype sugarcane plants were infected with *Colletotrichum falcatum* and were incubated at 25 ± 1 °C under light conditions (8 h dark + 16 h light) and data were recorded after 7, 15, and 21 days of infection (Figure 6). Transgenic plants over-expressing synthetic *SUGARWIN1* and *SUGARWIN2* genes were found to be better able to withstand pathogenic infection as compared with untransformed sugarcane plants (Figure 6).

## 3. Discussion

Sugarcane has a great potential for genetic engineering and has been engineered for various traits [23,24]. The fundamental reason is the cumbersome nature of sugarcane breeding [25]. Large and polyploid genome, very poor pollination and fertilization rate, above all, extraordinary lengthy breeding cycle are the major impediments in sugarcane breeding. On the other hand, genetic engineering is a robust and straightforward technique to produce novel crops that can fulfill the increasing demand for agricultural productivity [26]. The complex genome of sugarcane has been explored for the presence of numerous stress responsive genes. These endogenous plant genes are a valuable resource of transgenes to develop bio-safe transgenic plants with valuable traits. Establishment of a proficient in vitro callogenesis and subsequent regeneration system is a basic requirement for implementing genetic engineering techniques in plants. Compared with the dicots, sugarcane is more recalcitrant, non-responsive to regeneration and no leaf based regeneration system has been reported [27]. Sugarcane response to in vitro regeneration is highly genotype dependent [17]. We tested our selected genotype at different levels of hormones for callogenesis and regeneration. Hence, a proficient in vitro regeneration system was established in the selected genotype prior to its genetic transformation.

Recombinant *SUGARWIN2* has already been characterized to induce apoptosis in *C. falcatum* “the causal agent of red rot” [16]. Keeping in view the role of SUGARWIN proteins in red rot resistance, we proposed that over-expression of these genes may enable crop plants to perform better in the field under disease conditions. Regulatory sequences are of key importance, as far as transgene expression is concerned. Availability of a strong promoter, active in all cell types, is a desirable choice for the over expression of transgenes [28]. Maize ubiquitin (*Ubi-1*) promoter is contemplated as a better choice compared to *CaMV 35S* and *Adh1* for the constitutive expression of a transgene, particularly in monocots [29]. It has extensively been used for the transient as well as stable expression of transgenes in several monocots including wheat [30], maize [31], rice [32], sugarcane [33], and sorghum [34]. *SUGARWIN1* and *SUGARWIN2* genes were cloned in pUbiAB vector under maize ubiquitin promoter (*Ubi-1*) and *nos* terminator. The *bar* (bialaphos resistance) gene encoding phosphinothricin acetyltransferase provides resistance against phosphinothricin (herbicide), was used as selectable marker gene [35]. It was cloned in the same pUbiAB vector under *CaMV 35S* promoter and terminator, hence was physically linked with the recombinant *SUGARWIN* genes. Physical linkage between selection cassette and expression cassette is of pivotal importance to effectively transform plant cells. It increases the transformation efficiency by promoting growth of transformed cells in the selection media, thus growth of untransformed cells is suppressed. Sugarcane plants were co-transformed with both vectors containing *SUGARWIN1* and *SUGARWIN2* genes. Polymerase chain reaction analysis was carried out to confirm integration of the transgene. To reveal mRNA expression of the transgene, comparative expression analysis was performed and up-regulation of both of the genes (*SUGARWIN1* and *SUGARWIN2*) was observed in the engineered sugarcane plants. The confirmed transformants were challenged by *Colletotrichum falcatum* and were found to have higher level of resistance as compared with untransformed plants (Figure 6).

In sugarcane, pathogen enters through cane nodes, root primordia, growth ring, buds, and leaf scars [36,37]. Other entry sites include growth cracks, rootlets, and cut ends of setts at the time of sowing [6]. Mycelium starts to spread from cell to cell, followed by fungal invasion into stalk tissues. Pathogen produces a huge quantity of acid invertases, which hydrolyze plant’s sucrose into fructose and glucose for their consumption [38]. Fungi secretes cell wall degrading enzymes to depolymerize plant cell wall and also produces different toxins, as a result it induces signaling pathways leading to PCD (programmed cell death) [39,40]. SUGARWIN proteins are believed to be the crucial part of defense mechanism in sugarcane and have chitosanase as well as chitinase activity against pathogenic fungi. Hence, elevated level of these anti-pathogenic proteins in engineered sugarcane plants enabled them to uplift their level of tolerance against pathogen infection.

## 4. Materials and Methods

### 4.1. Callus Induction, Regeneration and Rooting

Sugarcane young leaf whorls were used for calli induction as release of phenolic compounds from all explants except young leaf whorls; it was a major problem in callus induction and regeneration. These phenolic compounds result in browning of the tissues. The young leaf whorls of healthy sugarcane plants were taken from the field, sterilized with methylated spirit or 70% ethanol, and upper hard layers of leaves were removed in laminar air flow cabinet. The apical meristematic segments were sliced into discs with 1.5–2.0 mm thickness and 60 to 95 mm^2^ area by sterilized scalpel. These slices were cultured onto callus induction medium comprising of Murashige and Skoog salts provided with 0.5 mg/L nicotinic acid, 1.0 mg/L thiamine HCl, 0.5 mg/L pyridoxin HCl, 2 mg/L glycine, 100 mg/L myoinositol, 30 g/L sucrose, and 3 mg/L 2,4-D (2,4-Dichlorophenoxyacetic acid) in petri plates and were kept in dark for 3 to 4 weeks [17].

Embryogenic calli were shifted to the regeneration media (RM). RM was comprised of Murashige and Skoog salts with 0.5 mg/L nicotinic acid, 1.0 mg/L thiamine HCl, 0.5 mg/L pyridoxin HCl, 100 mg/L myoinositol, 30 g/L sucrose, 2 mg/L glycine, and a variable quantity of 6-Benzylaminopurine (BAP). The media were congealed using 2.6 g/L gellan gum powder with a pH of 5.7. Then plates were kept in light (3000–4000 lux day intensity) at 25 ± 1°C. For multiplication and root induction, regenerated plants were shifted to the MS0 medium.

### 4.2. Development of Plant Expression Vectors

The nucleotide sequences of *SUGARWIN1* (GenBank: CA145787.1) and *SUGARWIN2* (GenBank: CA138924.1) genes were retrieved from NCBI and were modified, keeping in view the removal of unnecessary restriction sites with intact amino acid sequence. Moreover, necessary restriction sites (*Pst*I, *Xba*I, *Xho*I, *Spe*I, *Bam*HI, *Mlu*I, and *Kpn*I) were added at the 5′ and 3′ ends of gene sequences for further cloning into transformation vector and finalized sequences were synthesized commercially in pUC19 vector. Two independent expression cassettes were developed for both *SUGARWIN*1 and *SUGARWIN*2 genes by using same strategy. A *Spe*I/*Bam*HI *SUGARWIN* fragment was excised from pUC19 vector and was cloned in pUbiAB vector under maize ubiquitin (*Ubi-1*) promoter (a constitutive promoter) and *nos* terminator. The *bar* (bialaphos resistance) gene encoding phosphinothricin acetyltransferase provides resistance against phosphinothricin (herbicide), and was used as selectable marker gene. It was cloned in the same pUbiAB vector under *CaMV 35S* promoter and terminator and was physically linked with the recombinant *SUGARWIN* genes. This final transformation vector was confirmed by restriction analysis with various combinations of restriction endonucleases.

### 4.3. Genetic Transformation of Sugarcane

The optimized conditions for proficient calli induction and regeneration were used to engineer the *Saccharum* genome. Sugarcane genotype SPF-234 (red rot susceptible) was chosen for transformation, due to better agronomic performance and good response to callogenesis and regeneration. Biolistic^®^ PDS-1000/He particle delivery system (Bio-Rad, Hercules CA, USA) was used to bombard three to four week-old sugarcane calli with pUbiAB plasmid DNA-coated gold particles of 0.6 µm diameter by following the transformation protocol reported by Khan and Maliga [18]. Bombarded calli were kept at 25 ± 1 °C in dark for 48 h and were then cultured on regeneration medium provided with phosphinothricin and were kept in light (3000–4000 lux day intensity) at 25 ± 1 °C for regeneration. Then phosphinothricin-resistant shoots were shifted to MS0 medium for rooting. Explants were co-transformed with *SUGARWIN*1 and *SUGARWIN*2 genes.

### 4.4. Confirmation of Transgenic Plants by PCR Analysis

Total cellular DNA was isolated from leaf tissues of phosphinothricin-resistant plants as well as from untransformed sugarcane plants using miniprep method given by Spychalla and Bevan [41]. PCR (Polymerase chain reaction) was used to confirm the transgene integration [42]. Primers were designed and synthesized to amplify the 552 bp fragment of *bar* gene (*bar* primer pairs) and ~1 kb to amplify promoter-terminator region along with gene of interest (prom-term primer pair). *Taq* DNA polymerase was used with 100–300 ng of genomic DNA as a template. The *bar* gene was amplified with forward (5′-ATG GGC CCA GAA C-3’) and reverse (5´-TCA GAT CTC GGT G-3´) primers. Similarly, promoter-terminator region along with gene of interest was amplified by forward (CGTTCGTACACGGATGCGAC) and reverse (CAAGACCGGCAACAGGATTCA) primers. The thermal cycler profile was set as initial denaturation at 95 °C for 3 min, followed by 30 cycles at 95 °C for 1.5 min, 51 °C for 1.5 min, 72 °C for 2 min, and final extension at 72 °C for 10 min. Amplified fragments were observed by gel electrophoresis.

### 4.5. Transcriptomic Quantitative Analysis of Transgenic Sugarcane Plants Using qPCR

The technique of real time qPCR was employed to quantify the transcriptomic expression level of *SUGARWIN* genes in transgenic sugarcane plants in comparison with non-transgenic plants of the same genotype. For this purpose, total cellular RNA was isolated from around 100 mg of the leaf sample using GeneJET^TM^ plant RNA purification mini kit (ThermoFisher, Scientifc, USA). The isolates were treated with DNase I (ThermoFisher Scientifc, USA), followed by purification by the process of ethanol precipitation. For cDNA synthesis, the RevertAid First Strand cDNA synthesis kit (ThermoFisher Scientific, USA) was used following manufacturer’s protocol. An exact 1 µg of pre-quantified total cellular RNA prep was used in this process. The equal amounts of synthesized cDNAs were employed as templates to quantify the expression of *SUGARWIN* genes at transcriptomic level in transgenic as well as non-transgenic sugarcane plants. The qPCR reaction was performed using CFX96™ Real-Time PCR Detection System (Bio-Rad, Hercules CA, USA). The reaction conditions were normalized by using *Actin* and *GAPDH* as reference genes [43]. A 12 µL of reaction mixture was used comprising of 6.0 μL 2xSYBR^®^ Green PCR Supermix (BioRad, Hercules, CA, USA). The profile of thermal cycler was set as initial denaturation at 95 °C for 3 min, followed by 40 cycles at 95 °C for 90 s, 51 °C for 90 s, and 72 °C for 120 s. The primer specificity was determined by melt curve methods [44], which showed an exclusive peak, representing amplification of specific fragment and the absence of non-specific fragments or dimers. Primers flanking *SUGARWIN1* (Forward: 5′-AGG ATC GTG GAC CAG TGC AG-3′; Reverse: 5′-CGT TGA GGT GTC CCA TCT G-3′) and *SUGARWIN2* (Forward: 5′-GTG AGG ATC GTG GAC CAG T-3′; Reverse: 5′-CTG GTA GTT GAC GGT GAG G-3′) were run against cDNA of transformed and untransformed sugarcane plants.

### 4.6. Fungal Bioassays

The resistance level of sugarcane plants against *Colletotrichum falcatum* was evaluated by fungal bioassays. In vitro grown transgenic sugarcane plants were selected for this assay. Two PDA agar blocks (0.5 cm^2^) of *Colletotrichum falcatum* with virtuous mycelial growth were placed in the glass jars having plants. The inoculated plants were incubated in the light at 25 ± 1 °C under light conditions (8 h dark + 16 h light) and the morphological symptoms (at 7, 15 and 21 days of post infection) were observed to see the impact of fungal pathogen on plant growth [1]. In vitro grown wild type sugarcane plants were used as control. The experiment was run in triplicate.

## 5. Conclusions

Endogenous sugarcane genes (*SUGARWIN1* and *SUGARWIN2*) were over-expressed in red rot susceptible sugarcane genotype SPF-234. Phosphinothricin-resistant plants were confirmed through PCR for transgene integration, whereas transgene expression was assessed through real-time qPCR. Expression of both of the antifungal proteins appeared to be elevated under the control of maize ubiquitin promoter. Further, fungal bioassays revealed that transgenic plants are better tolerant to *Colletotrichum falcatum,* as compared with untransformed plants.
